# Fiber-Reinforced Asphalt Mixture Design on Anti-Skid Surfacing for Field Testing High-Speed Vehicles on Pavements

**DOI:** 10.3390/ma16020549

**Published:** 2023-01-05

**Authors:** Hao Liu, Yunyu Li, Jixin Li, Feng Wang, Longfan Peng, Chao Li, Tianlei Wang, Juntao Lin

**Affiliations:** 1 China Construction Second Engineering Bureau Ltd., Beijing 100176, China; 2 State Key Laboratory of Silicate Materials for Architectures, Wuhan University of Technology, Wuhan 430070, China; 3 School of Transportation and Logistics Engineering, Wuhan University of Technology, Wuhan 430070, China; 4 Faculty of Engineering, China University of Geosciences (Wuhan), Wuhan 430074, China

**Keywords:** fiber, asphalt mixture, skid resistance, vehicles testing field pavement, surface texture

## Abstract

Fiber can absorb asphalt binder and therefore reinforce and stabilize the asphalt mixture structure and also prevent the asphalt from the leaking, which occurs in the process of mixing and transport. In this study, three kinds of fiber (polyester fiber, polypropylene fiber, and lignin fiber) are used to evaluate the relationship between the fiber types and mechanic performance of SMA-13 fiber asphalt mixture, which is specially designed for field tests of high-speed vehicles on pavements. The micro-surface characteristics of fiber and aggregates were studied by SEM and image analysis. Marshall stability and splitting strength were used to measure the properties of the asphalt mixture. In addition, a field test, including measures for curve-section edge, curve-section center, straight-section edge, and straight-section center, was conducted to evaluate the skid resistance of the high-speed vehicles that test field pavement. The results show that the Marshall stabilities of asphalt mixture with three kinds of fibers have been improved, whereas the stability of asphalt mixture prepared by polypropylene fiber and polyester fiber particularly increased before immersion. Among the three kinds of fiber asphalt mixtures, the polyester fiber asphalt mixture has enhanced water susceptibility. Skid resistance in the field test indicated that high skid resistance and good surface-texture depth were achieved.

## 1. Introduction

Asphalt pavement is widely used due to its good functional performance, including fast traffic opening, easy maintenance, and renewable utilization [1,2]. However, asphalt in China is mainly derived from paraffin-based crude oil that is sensitive to temperature, resulting in the temperature sensitivity of asphalt pavement being high [3]. Additionally, asphalt pavement tends to present various problems with the use of ordinary asphalt [4,5,6,7]. For example, asphalt pavement is prone to oil, and soft and heavy loads easily form permanent deformations, such as rutting and pushing in summer, as well as brittle cracking in winter. Consequent to these deformations, the stability of asphalt is regarded as particularly important. In order to solve the above problems, a lot of experimental research has been carried out at home and abroad [8,9]. To improve surface performance, fiber has been added in many ways to enhance the stability of asphalt mixtures, which can effectively improve the comprehensive performance of asphalt slurry and its mixture, improve the quality of asphalt pavement, prolong its service life, and reduce the amount of maintenance [10,11,12,13]. The asphalt mixture is mainly composed of aggregate and asphalt slurry, which includes asphalt and mineral powder. Although the content of the asphalt slurry in asphalt mixture is small, it plays a key role in the quality of the asphalt mixture [14,15,16,17]. Fiber asphalt slurry is a mixture of asphalt, fiber, and mineral powder. Fiber can absorb asphalt, which can reinforce and stabilize the asphalt mixture structure and also prevent the asphalt leaking, which occurs in the process of mixing and transport [11,18,19,20].

Fiber has been used as a reinforcing material in road construction since the 20th century, and it can be classified as organic, metallic, mineral, glass, or synthetic (polyester, polypropylene, and aramid). In 1960, Davis from the University of Toronto conducted the first systematic study on asbestos fiber for road use [21]. After that, studies on asbestos-fiber-modified asphalt mixture continued to be carried out, while asbestos was banned due to its huge harm to the environment and great impact on human health. In 1984, lignin fiber replaced asbestos fiber as the stabilizer in SMA mixtures, and thus lignin-fiber-modified asphalt mixture research was carried out. However, there are also certain limitations of using lignin fiber, such as its characteristic of easy water absorption, and that it easily decomposes coke, which pollutes asphalt [21]. The application of polymer fibers began in the 1980s with a patent by DuPont in the United States, which applied a specially formulated reinforcing fiber made of polyester material to an asphalt mixture. Additionally, the research of Freeman showed that polyester fiber could significantly improve the road performance of asphalt mixtures in 1989 [21]. Polyester fiber was added to the asphalt mixture after the surface, along with an anti-aging treatment that can enhance the bonding strength between the asphalt and aggregate to make the connection closer [22]. Meanwhile, the fiber can not only improve the low-temperature stability and high-temperature stability but can also optimize the mechanical properties of asphalt mixture, which increases the service life of the pavement [23,24]. The application of fiber in asphalt mixture has been studied by many scholars, and the influence of fiber on asphalt properties depends on the properties of the fiber itself. Yu [25] compared the effects of basalt fiber, lignin fiber, and polyester fiber on the performance of asphalt mixtures and found that basalt fiber can improve the low-temperature performance of asphalt mixtures more than the other two kinds of fiber. It has also been shown that the fiber can effectively prevent the formation and development of asphalt slurry deformation and improve the ability to resist shear degeneration.

Fibers can be added to asphalt or directly to asphalt mixtures, and there are generally two methods of adding fibers to asphalt mixtures, including the dry method and the wet method [18,21,26]. The dry method is to mix the fiber and mineral evenly and then add asphalt to the mix to form an asphalt mixture. The wet method can be divided into two types according to the different stages of fiber addition. In the first method, the fiber is added to the asphalt and stirred evenly through a high-speed shear mixer and then added to the mineral material before being mixed to form the asphalt mixture. However, this method requires a high-speed shear mixer, the mixing uniformity effect is not significant, and the fiber oil absorption leads to errors in asphalt dosage calculations compared with the dry method, and thus there are no advantages. In the second method, asphalt and minerals are mixed before adding fibers. Among the three addition processes, the dry method, where fiber agglomeration is the weakest, is relatively simple. Furthermore, the dry method can better distribute the fiber in the mixture; therefore, the dry process is often used in fieldwork using fiber asphalt mixtures.

Therefore, in this study, three kinds of fiber (polyester fiber, polypropylene fiber, and lignin fiber) are used to design asphalt mixtures, and polyester fiber was used to design anti-skid surfacing materials for field tests of high-speed vehicles on pavements, which always need high-quality skid resistance, good surface texture, and strength capacity. Firstly, the morphology and microstructure of the three kinds of fiber were characterized by image analysis and SEM test. Secondly, fracture strength and melting point were studied to observe the basic properties and high-temperature properties of fiber, and a dry method was used to prepare the fiber asphalt mixture. Thirdly, based on the laboratory test, the impact of fiber types on the enhancing effect of asphalt mixture was studied, and the effect of fiber on the skid resistance of asphalt pavement was then evaluated in the field.

## 2. Materials and Properties

### 2.1. Raw Materials

#### 2.1.1. Asphalt

SK Speedway modified asphalt was used for high-speed vehicles testing for field asphalt mixtures design. Table 1 shows the asphalt physical tests, for instance, penetration, softening point, and ductility tests were conducted for determining basic performance. It can be found from Table 1 that the penetration, ductility, and softening point of the used modified asphalt binder all meet the specifications.

#### 2.1.2. Aggregate

Basalt was selected as aggregate in this study. In order to reduce the error caused by aggregate size and enhance the repeatability of the following research test, the aggregate was divided into different particle size groups; then, the performance test was carried out. The basic performance indicators of coarse and fine basalt aggregate are shown in Table 2 and Table 3.

#### 2.1.3. Fibers

As an additive of the specially designed high-speed vehicles testing the field asphalt mixture, the performance characteristics of fiber itself have a significant impact on its mixture enhancement effect. The various performance indexes of fiber will have an obvious influence on road performance. The various performance parameters of the three used fibers are shown in Table 4.

It can be seen from the fracture strength in the table that polyester fiber has the highest strength. Polyester fiber, lignin fiber, and polypropylene fiber rank from the largest to the smallest in terms of length–diameter ratio. If the fiber length–diameter ratio is too small, it cannot reinforce and toughen the properties of the final asphalt mixture, thus it is difficult to improve the strength of asphalt mixture. While the length–diameter ratio is too large, fiber is easy to cluster in the mixing process and difficult to disperse, resulting in the strength of asphalt mixture not considerably increasing. Furthermore, the melting temperature of the fibers should be higher than that of the mixing temperature of the asphalt mixture; thus, the three kinds of fibers selected in this paper meet the requirements.

## 3. Research Methodologies

**Figure 1:**
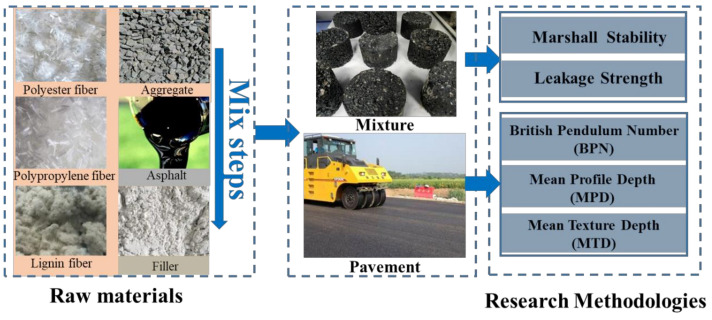
Research methodologies.

The dry method was used to add fiber into the asphalt mixture in this study. Firstly, three kinds of fibers were mixed with coarse and fine aggregate. Then, asphalt binder and filler were added successively. Figure 1 shows the mixture preparation process. According to the specification requirements, the mixing and compaction temperature of the hot mixture should be increased by 10 °C after adding fiber. Thus, the heating temperature at each stage of the mixture or pavement preparation is selected as follows: heat asphalt binder to 160 °C, heat aggregate to 170 °C, heat fiber to 160 °C, mixing at 160 °C, and compacting at 150 °C. To make the fiber as evenly dispersed as possible and prevent agglomeration, the total mixing time of the dry method should be longer than that of traditional mixing. The actual operation is to mix coarse and fine aggregate evenly for 30 s, while the time for mixing fiber is 60 s, asphalt is 90 s, and filler is 90 s. After the internal temperature of the mixing drops to 150 °C, the specimen is compacted 75 times on both sides to form Marshall specimens of asphalt mixtures.

Marshall stability and splitting strength were used to measure the properties of the asphalt mixture. The splitting test used three groups of specimens and each group had three specimens. Meanwhile, the test temperature was maintained at 25 °C, and the loading displacement rate of 50 mm/min was used. The first group was used for comparison, while the other two groups were treated at water conditions with 3500 cycles of hydrodynamic pressures. All specimens were used for the measurement of splitting strength.

**Figure 2:**
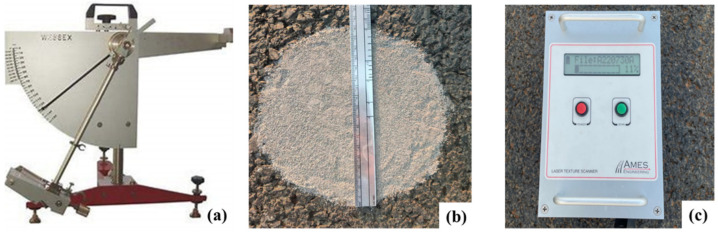
Portable pendulum tester (**a**), sand patch test (**b**), Laser Texture Scanner (**c**).

In addition, four locations from the high-speed-vehicles testing field were selected in this study to evaluate the skid resistance of the designed anti-skid surfacing mixture, including curve-section edge (CE), curve-section center (CC), straight-section edge (SE), and straight-section center (SC). The detection parameters include BPN (British pendulum number) value, MTD (mean texture depth), and MPD (mean profile depth), and the three involved equipment are shown in Figure 2. Figure 2a shows a portable pendulum tester, which measures the BPN value to evaluate the skid resistance of asphalt pavement with moisture. Figure 2b shows a circle enclosed by standard sand, where the sand patch test is widely used to detect the MTD of asphalt pavement surface, and Figure 2c shows a LTS9400 (Laser Texture Scanner 9400, Ames Engineering, Iowa State, America), which can obtain the MPD of a specific area. The skid resistance of the pavement was detected before opening to traffic [27].

## 4. Results and Discussions

### 4.1. Fiber Micro-Characteristics

**Figure 3:**
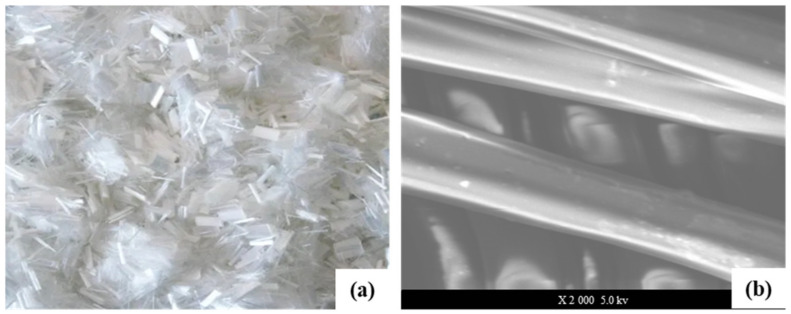
Polyester fiber (**a**), microstructure of polyester fiber (**b**).

It can be seen from Figure 3a that polyester fiber is a synthetic fiber prepared by spinning after the polycondensation of organic diacid and dialcohol, which is milky white with mercerization, moderate elongation, high modulus, and good chemical stability. To further identify the surface structure of polyester fiber and analyze its internal factors that might affect the performance of asphalt mixture, SEM was used, and the corresponding microstructure is shown in Figure 3b.

**Figure 4:**
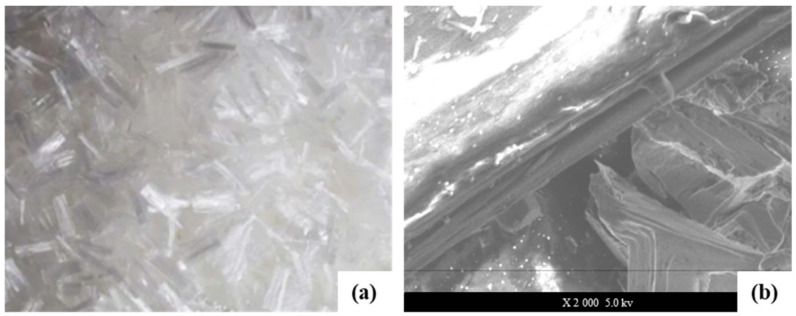
Polypropylene fiber (**a**), microstructure of polypropylene (**b**).

Polypropylene fiber is polymerized from copolymer monomer and acrylonitrile to produce polyacrylonitrile resin; then, it is dissolved in a solvent to form a spinning solution. Finally, it is spun by the dry method or wet method; thus, fiber is obtained through a variety of complex processes. The high thermal stability of polypropylene fiber can withstand higher temperatures up to 190 °C, with strong acid resistance, strong alkali resistance, and weak thermal conductivity. It can play a significant role in the structural stability. Meanwhile, it can effectively inhibit the shrinkage deformation induced by drying and temperature differences and improve anti-cracking properties to a certain extent. The morphology and microstructure of polypropylene fiber are shown in Figure 4.

**Figure 5:**
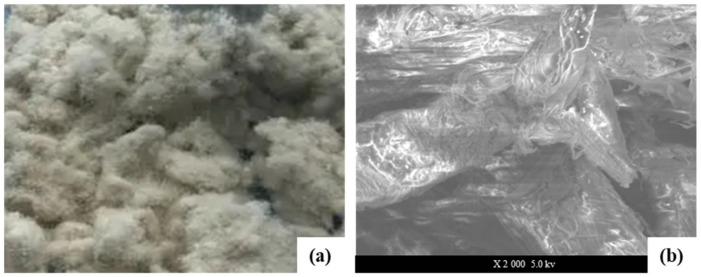
Lignin fiber (**a**), microstructure of lignin fiber (**b**).

Lignin is an amorphous three-dimensional mesh aromatic polymer, which consists of a carbon–carbon bond and an ether bond linked to the structure of phenylpropane. Each polymer unit contains hydroxyl, methoxy, and carbonyl groups. Lignin fiber is a kind of plant fiber that is processed chemically or mechanically from wood. It is widely used because of its strong oil absorption ability, good chemical stability, and low price. Lignin fiber has a large and rough surface area, as well as high heat resistance, and is widely used in asphalt mixtures to absorb and stabilize asphalt. As shown in Figure 5a, the lignin fiber is gray flocculent without dispersing in clusters, clumped into fragments by hand, and is uniform in length and thickness. Figure 5b shows that multiple lignin fibers have uniform diameters and interweave with each other, and they are also not easy to disperse.

### 4.2. Anti-Skid Mixture Design

#### 4.2.1. Aggregate Gradation Design

SMA-13 gradation is adopted in this study to compare the effects of polyester fiber, polypropylene fiber, and lignin fiber on the field-test performance of asphalt mixtures using high-speed vehicles. Firstly, three gradations (gradation A, gradation B, and gradation C) of fiber-modified SMA-13 were determined, and the passing rates of the 4.75 mm sieve were 23.9%, 26.8%, and 29.8%, respectively. The compositions of the three aggregate gradations are shown in Table 5.

Voids in coarse aggregate under the dry-rodded condition (VCA_DRC_) of the three gradations were measured. The initial test specimens were made with the ratio of asphalt aggregate at 6.4%, with 50 times compaction on both sides. Voids in the coarse aggregate of a compacted mix (VCA_mix_), as well as voids in mineral aggregate (VMA), were measured. The gradings were then determined based on the condition that VCA_mix_ was less than VCA_DRC_, and that VMA was not less than 17%. The results are shown in Table 6 and Table 7.

**Figure 6:**
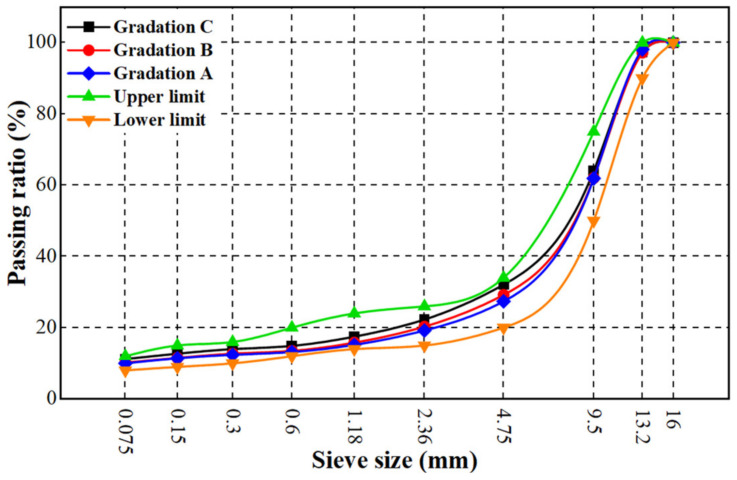
Grading curves of the SMA-13 mixtures.

It can be seen from Table 6 and Table 7 that the volume indexes of gradation C meet the requirements, while the air voids of gradation A and B are not satisfied. Therefore, gradation C was chosen as the design grade in the following research. Figure 6 shows the grading curve.

#### 4.2.2. Optimal Asphalt Content Design

**Figure 7:**
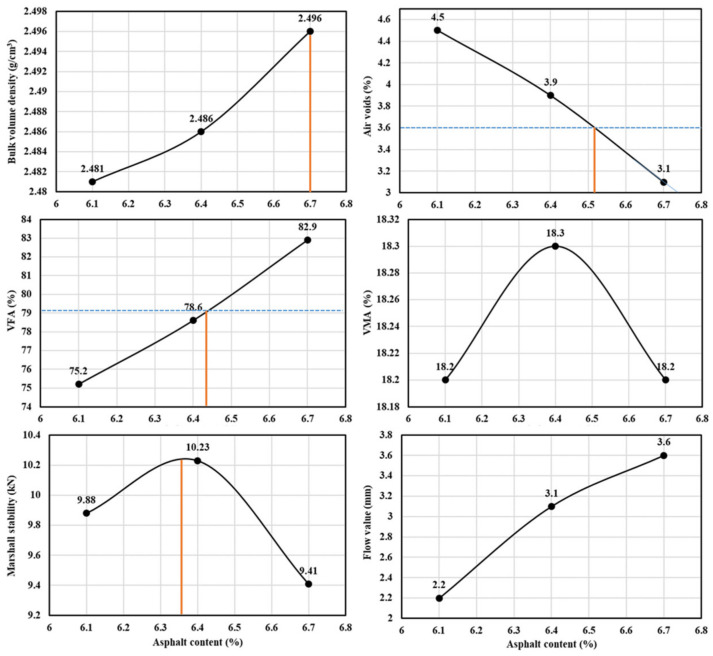
Marshall test results with different asphalt content.

After the aggregate gradation was fixed, the Marshall compaction test of the designed gradation was carried out with three asphalt contents of 6.1%, 6.4%, and 6.7%, and the optimum asphalt content (OAC) was determined based on the performance of mixture with these three different asphalt contents. With asphalt content as the horizontal coordinate and various indexes of the Marshall test as the vertical coordinate, the relationship of the Marshall test results is drawn, and the results are shown in Figure 7.

On the relation curve, the asphalt content a_1_ = 6.7% corresponding to the maximum density was taken. The asphalt content relative to the maximum Marshall stability a_2_ = 6.36%, asphalt content corresponding to design air voids ratio a_3_ = 6.52%, and the median voids filled with asphalt (VFA)-relative asphalt content a_4_ = 6.42%. OAC_min_ = 6.73 was obtained when the lower limit of the design air voids was 3%. OAC_max_ = 6.85 was obtained when the design saturation reached 85%. Then, the optimal asphalt content can be calculated according to the following formulas, where OAC is 6.7%.

(1) $${\mathrm{OAC}}_{1}\;{=\;(a}_{1}{\;+\;a}_{2}{\;+\;a}_{3}{\;+\;a}_{4})/4$$

(2) $${\mathrm{OAC}}_{2}{\;=\;(\mathrm{OAC}}_{\min}{\;+\;\mathrm{OAC}}_{\max})/2$$

(3) $${\mathrm{OAC}=(\mathrm{OAC}}_{1}{\;+\;\mathrm{OAC}}_{2})/2$$

### 4.3. Fiber Influence Mechanism

#### 4.3.1. Marshall Stability

**Figure 8:**
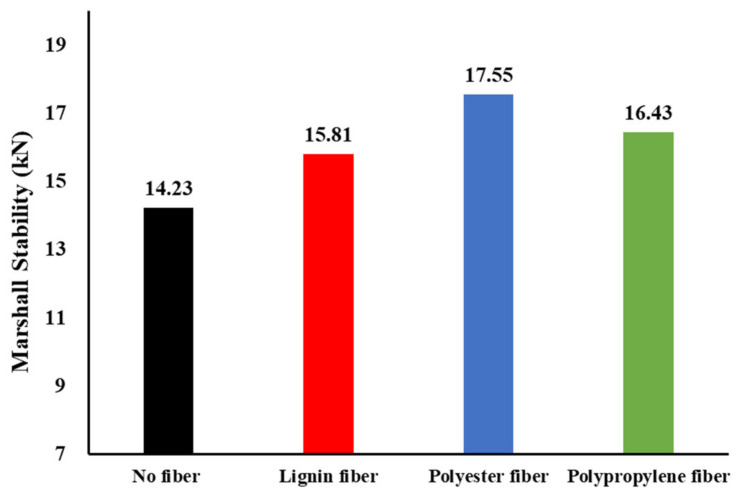
The results of Marshall stability.

Figure 8 shows the results of the Marshall stability of the asphalt mixtures. It can be seen that the Marshall stabilities of the asphalt mixtures with three kinds of fibers are improved, whereas, the stability of the asphalt mixture prepared by polypropylene fiber and polyester fiber particularly increased before immersion. This is attributed to polypropylene fibers and polyester fibers being less dense than lignin fibers; thus, more fiber with the same adding mass ratio can form a denser fiber network in an asphalt mixture, resulting in a greater ability to transfer and disperse loads.

#### 4.3.2. Moisture Susceptibility

The effect of fiber on the moisture susceptibility of asphalt mixture was evaluated by the splitting test and immersion Marshall test. The purpose of the splitting test is to evaluate the ability of fiber asphalt mixture to resist water damage after dynamic scouring. The splitting test conducts an analysis of the dynamic water scour effect on asphalt mixture under specified conditions to determine the strength ratio of the splitting failure of asphalt mixture before and after water damage. The splitting strength ratio is calculated using the following formula:

(4) $$\mathrm{TSR}\;=\;\frac{{\mathrm{RT}}_{2}}{{\mathrm{RT}}_{1}}\;\times \;100\%$$

where:

TSR is the splitting strength ratio (%);

RT_1_ is the splitting strength of specimens without dynamic water scour;

RT_2_ is the splitting strength of specimens with dynamic water scour.

The purpose of the immersion Marshall test is to evaluate the ability of the fiber asphalt mixture to resist aggregate spalling with water damage. We used an asphalt mixture Marshall test instrument for the immersion Marshall test, and the following formula was used for residual stability:

(5) $${\mathrm{MS}}_{0}\;=\;\frac{{\mathrm{MS}}_{1}}{\mathrm{MS}}\;\times \;100\%$$

where:

MS_0_ is the residual stability of the specimen in water (%);

MS is the Marshall stability before immersion (kN);

MS_1_ is the Marshallian stability after being immersed in water for 48 h (kN).

**Figure 9:**
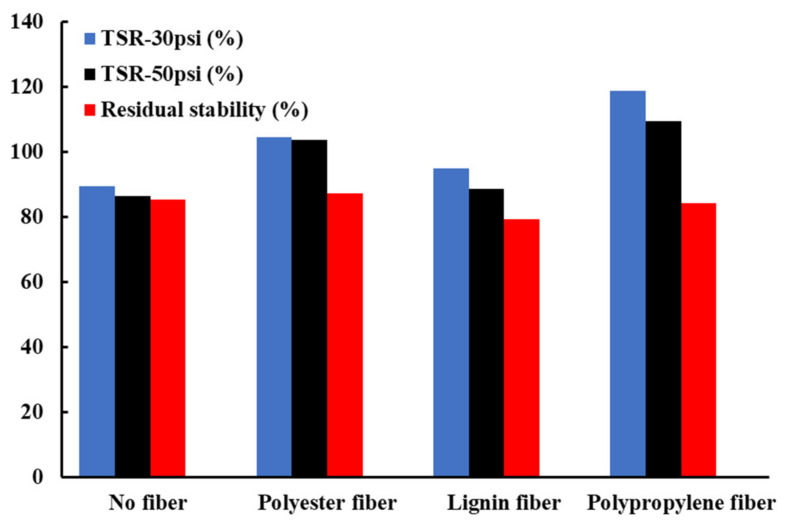
The relationship between the water susceptibility and fiber types.

The results of the residual stability and splitting strength ratio tests of asphalt mixtures with three different fibers and without fiber are shown in Table 8. According to the data in the table, the connection between the susceptibility to asphalt mixture and fiber types can be obtained. It can be seen from Figure 9 that the splitting strength of asphalt mixture decreases with the increase in dynamic scouring pressure, among which the splitting strength of polypropylene fiber asphalt mixture is the largest. Additionally, a variation in the splitting strength of polypropylene fiber asphalt mixture is the largest when the dynamic scouring pressure increases from 30psi to 50psi, while the change in polyester fiber is the least considerable. However, only the residual stability of polyester fiber increases, which meets the requirements of the specification by no less than 85%, while the residual stability of the asphalt mixture prepared by lignin fiber and polypropylene fiber decreases. Therefore, considering the residual stability and the splitting strength ratio after hydrodynamic scouring, only the polyester fiber asphalt mixture has enhanced water susceptibility among the three kinds of fiber asphalt mixtures.

### 4.4. Skid Resistance of Testing Field

#### 4.4.1. BPN and MTD

According to the above results of the moisture susceptibility to the fiber asphalt mixture, the field-test section of the polyester fiber pavement was then studied. In this paper, the traditional test method of skid resistance, including the portable pendulum tester and sand patch test, were conducted. Five points were selected for each test location, and the BPN20 of the final standard temperature of 20 °C of each point was measured. The texture depth of the asphalt mixture was calculated according to the formula below, which is accurate to 0.01 mm, where TD is the texture depth of asphalt mixture (mm), V is the volume of sand (25 cm^3^), D is the diameter of flattened sand (mm), and the final average value of the five points is MTD.

(6) $$\mathrm{TD}\;=\;\frac{1000\;\times \;V}{{\pi \;\times \;D}^{2}/4}=\frac{31831}{D^{2}}$$

**Figure 10:**
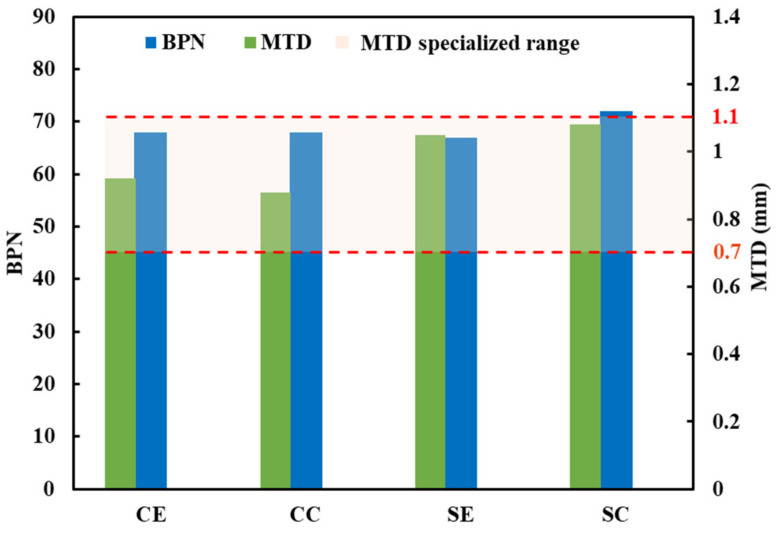
The results of BPN value and MTD.

The results of BPN value and MTD are shown in Figure 10, which shows that the BPN value of CE is the same as that of CC, and is similar to SE, while the BPN value of the SC is significantly higher than that of other areas. The MTD all meet the specified requirements, which range from 0.7 mm to 1.1 mm. The MTD of the SC was the largest, and the MTD of the curve was obviously smaller than that of the straight road, which may be because the driver is prone to abnormal acceleration when the vehicle drives on straight sections for a long time; therefore, high skid-resistance requirements are needed for straight-section pavement [28]. Furthermore, the transverse slope was added to the design process of the curve-section pavement, which can counteract the centrifugal force on the vehicle.

#### 4.4.2. Mean Profile Depth

**Figure 11:**
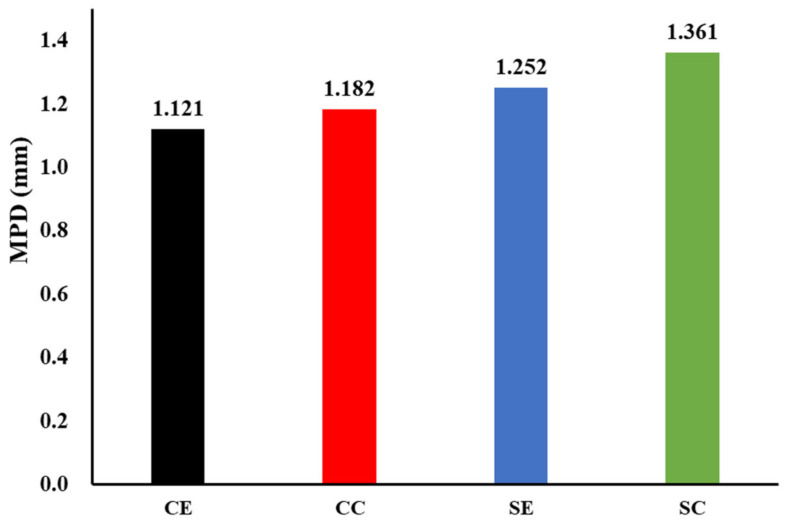
The results of MPD.

The LTS9400 has a scanning distance of 0.01784 mm between two laser spots, a maximum resolution of 0.02469 mm in width, a range of 0–2835 scanned lines, and a maximum scanning area of 100 mm × 70 mm. Ten scan lines were used for four different areas several times to obtain mean profile depth, and 600 lines scanned the CC and SC to obtain 3D models since the center of the pavement is where vehicles often drive. The results of the MPD are shown in Figure 11, which shows that the MPD at the straight area is obviously larger than that at the curve, while the MPD at the SC is the largest, which has the best skid-resistance performance [29].

**Figure 12:**
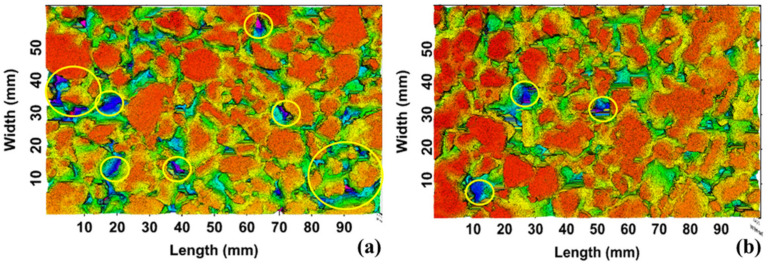
The 3D models of pavement: (**a**) CC, (**b**) SC.

Figure 12 shows the 3D model of the pavement surface obtained by laser scanning, Figure 12a is the curve section, and Figure 12b is the straight section. The red area represents the positive texture, while the blue part represents the negative texture. The darker the red color is, the greater the MPD will be. It can be seen from Figure 12a that the blue areas in the curve area are significantly more than those in the straight pavement, which are also concentrated while appearing in straight pavement as islands. Additionally, the red area in the straight area is obviously more than that in the curve area. This indicates that there are more negative textures in curve pavement than in the straight, and the amount of positive textures in the straight is significantly greater than that in the curves. Furthermore, it has been proved that the contribution of positive textures to anti-skid performance is greater than that of negative textures [30]. Therefore, it can be concluded that the original skid resistance of straight pavement is better than that of curved pavement, which is consistent with the conclusion reached by the conventional skid-test method.

## 5. Conclusions

This study used three kinds of fibers to design asphalt-mixture-based anti-skid surfacing for field testing high-speed vehicles on pavements. Materials characteristics, anti-skid surfacing mixture design, and field pavement verification were conducted and discussed. Based on the discussed experimental results, the following conclusions are drawn:

1. Fiber can be used to improve the Marshall stabilities of asphalt mixture for field testing with high-speed vehicles, while polypropylene fiber and polyester fiber particularly provided the best modification results in terms of Marshall stability;
2. According to the study regarding hydrodynamic treatment, polyester fiber can be used to improve the water susceptibility of asphalt mixture in terms of residual stability and splitting strength ratio;
3. The results of field tests show that the maximum mean profile depth can reach up to 1.361 mm, while the maximum BPN reaches 70;
4. Field verification indicates that there are more negative textures in curve sections than in straight sections, and the amount of positive textures in straight sections is significantly greater than that in curve sections. The original anti-skid performance in straight sections is better than that in curve sections.

## Figures and Tables

**Table 1:** Basic performance indicators of SK Speedway modified asphalt.

Properties	Units	Test Value	Specification
Penetration at 25 °C	0.1 mm	58.6	50–60
Ductility at 5 °C	cm	23.8	≥20
Softening point	°C	79.1	≥60
Flash point (COC)	°C	365	≥230
Kinematic viscosity at 135 °C	Pa.s	2.2	≤3
Solubility (trichloroethylene)	%	99.6	≥99

**Table 2:** Basic performance indicators of coarse basalt aggregate.

	Size	11–18 mm	6–11 mm	3–6 mm	Specification
Properties					
Specific gravity (g/cm^3^)		2.953	2.961	2.934	ASTM C127
Silt content (%)		0.4	0.5	0.3	T0310-2005
Water absorption (%)		0.48	0.45	0.40	T0307-2005
Adhesion (grade)		5	5	-	T0616-1993

**Table 3:** Basic performance indicators of fine basalt aggregate.

Properties	Units	0–3 mm	Requirements	Specification
Specific gravity	-	2.650	≥2.5	T0328-2005
<0.075 mm content	%	0.2	≤1	T0314-2005
Sand equivalent	%	64	≥60	T0334-2005

**Table 4:** General properties of the used fibers.

Properties	Polyester Fiber	Polypropylene Fiber	Lignin Fiber
Length (mm)	4–40	3–19	0.5–20
Diameter (μm)	24	32	14
Fracture strength (Mpa)	508	450	298
Moisture content (%)	1	0.8	5
Melting point (°C)	260	172	210

**Table 5:** The mass percentage of three gradations of mixture through the sieve.

Gradation Types	Percentage of Mass Passing through the Following Screen (mm) (%)									
	16.0	13.2	9.5	4.75	2.36	1.18	0.6	0.3	0.15	0.075
Gradation A	100.0	98.1	61.9	27.4	19.2	15.2	13.2	12.4	11.4	10.0
Gradation B	100.0	97.2	62.0	29.2	20.3	15.8	13.5	12.7	11.5	10.2
Gradation C	100.0	97.3	64.0	32.1	22.3	17.5	14.9	14.0	12.7	11.2

**Table 6:** The results of VCA_DRC_.

Grain Grading	Pounding Density (t/m^3^)	Passing Ratio by 4.75 mm Sieve (%)	Gross Density (g/cm^3^)	Voids in Coarse Aggregate under Dry Rodded Condition VCA_DRC_ (%)
A	1.726	23.9	2.933	41.2
B	1.695	26.8	2.933	42.2
C	1.657	29.8	2.933	43.5

**Table 7:** Gradation volume analysis.

Grain Grading	Bitumen Aggregate Ratio (%)	Specimen Gross Density (g/cm^3^)	Maximum Theoretical Relative Density (g/cm^3^)	Air Voids (%)	Voids in Mineral Aggregate VMA (%)	Voids Filled with Asphalt VFA (%)	Coarse Aggregate Skeleton Clearance Rate VCA_mix_ (%)
A	6.4	2.472	2.585	4.4	18.9	76.8	40.8
B	6.4	2.470	2.579	4.2	18.7	77.5	40.9
C	6.4	2.479	2.572	3.6	18.2	80.1	40.7
requirements	-	-	-	3–4	≥17.0	75-85	≤VCA_DRC_

**Table 8:** General moisture resistance properties of the used fibers.

Fiber Types	TSR-30psi (%)	TSR-50psi (%)	Residual Stability (%)
No fiber	89.22	86.33	85.2
Polyester fiber	104.31	103.45	87.1
Lignin fiber	94.70	88.64	79.1
Polypropylene fiber	118.52	109.26	84.1
